# Does That Thumb Trigger Finger Release Have You Feeling “Nerve”-ous?

**DOI:** 10.7759/cureus.116484

**Published:** 2026-09-18

**Authors:** Sumit S Patel, Maxwell Albiero, Raymond Bayer, Richa Gupta, Mayron Lichterman, David L Mayor

**Affiliations:** 1 Orthopaedic Surgery, Western Michigan University, Kalamazoo, USA

**Keywords:** digital nerve, orthopaedic hand surgery, radial digital nerve to thumb, thumb trigger finger, ulnar digital nerve to thumb

## Abstract

Purpose

The purpose of this investigation is to determine anatomical variations in the specific location of the radial and ulnar digital nerves to the thumb crossing the flexion crease. Understanding the precise location of these nerves is crucial for surgical interventions, such as in thumb trigger finger release, where the nerves are in danger of transection.

Methods

Twenty-one soft-embalmed cadaveric hands were dissected. ImageJ (NIH, Bethesda, MD) was used to determine the distance the ulnar and radial digital nerves to the thumb cross at the proximal flexion crease from the radial aspect of the crease.

Results and conclusions

The radial digital nerve crossed the crease at 8.38 ± 3.38 mm from the radial aspect of the crease. The ulnar digital nerve crossed the proximal flexion crease of the thumb approximately 16.35 ± 3.80 mm from the radial aspect of the crease. On average, the radial digital nerve crossed at 36.4% ± 9.4% of the distance between the radial and ulnar portions of the flexion crease, while the ulnar digital nerve crossed at approximately 72% ± 8.2%. The radial digital nerve crossed at the radial third of the crease in 43.0% of specimens and in the middle third in 57.0% of specimens. The ulnar digital nerve crossed at the ulnar third of the crease in 81.0% of specimens and in the middle third in 19.0% of specimens.

## Introduction

The radial and ulnar digital nerves to the thumb are important functional units of the thumb, contributing to the sensory innervation of the respective divisions of the thumb. These nerves are significant in various aspects of hand function, emphasizing the need for both anatomical understanding and application during clinical practice [1]. Their pathways, particularly as they traverse the flexion crease, are of important clinical significance in the context of surgical procedures involving the hand to avoid iatrogenic injury.

Digital nerves are responsible for relaying sensory information from the radial and ulnar sides of the thumb, which are regions that are important for tactile perception [2]. The clinical relevance of the radial digital nerve becomes particularly pronounced in surgical interventions targeting the hand and thumb [3]. The A1 pulley of the thumb is a fibrous band of tissue that holds the flexor tendon close to the bone, maximizing the tendon's force production and efficiency [4]. Tendon swelling or pulley thickening can cause the condition known as trigger finger, where the finger can be painful and have mechanical symptoms like catching, clicking, or even locking [5]. Surgical release of the A1 pulley can help resolve these symptoms. Trigger finger releases of the A1 pulley of the thumb require a meticulous understanding of the radial digital nerve’s course, variations in its pathway, and its relationship with adjacent structures [6]. Often less discussed, the ulnar digital nerve crosses in a similar fashion along the flexion crease with variable anatomy. An oversight in recognizing and respecting the anatomical intricacies of the digital nerves can lead to increased risk of iatrogenic nerve injuries, potentially compromising the patient’s sensory function [7].

Numerous studies have emphasized the vulnerability of digital nerves during surgical procedures [8-10]. Classically, the radial digital nerve has been described as being most at risk during thumb trigger releases. Although relatively uncommon, injuries to the radial digital nerve are devastating and create high morbidity outcomes for the patient [11,12]. Guler et al. have described two patients with a 5.7% rate of digital nerve injury in percutaneous trigger thumb release and advocate for open release due to the rate of complication [13]. Dębski et al. have underscored the importance of reconstructing digital nerves after injury to enhance postoperative sensory outcomes [7]. The depth of the nerves in relation to the digital creases of the index finger and the course of the digital nerves to the thumb have been described by Wallace and Coupland, but specific values were not given for distance from anatomic landmarks of the thumb [14]. Additionally, Bas and Kleinert as well as Zenn et al. have contributed valuable insights into the anatomical variations of digital nerves in the hand, laying the foundation for a deeper understanding of the thumb’s digital nerves’ course and its implications for surgical practice [15,16].

The goal of this investigation is to explore the variations in the course of the radial and ulnar digital nerves to the thumb, particularly as each of them crosses the proximal thumb flexion crease. Our research seeks to provide a foundation for improved surgical planning, ultimately minimizing the risk of iatrogenic injuries and optimizing patient outcomes in hand surgery. This article was previously presented as a poster presentation at the 2025 AAOS Annual Meeting from March 10 to 14, 2025.

## Materials and methods

This anatomic study was performed on 21 soft-embalmed, cadaveric hands in Kalamazoo, Michigan. These were from 21 unique bodies, and no antimeres were utilized. The hands were dissected by three orthopedic surgery residents in an atraumatic fashion to minimize neurolysis and movement of the nerves. Hands with any form of prior hand surgery or those cadavers that were not well maintained were excluded. Thumb tacks were used to mark each end of the proximal flexion crease of the thumb following dissection. Images were then taken of each hand using an iPhone 15 Pro (Apple Inc., Cupertino, CA) equipped with a 48-megapixel camera (Figure 1).

**Figure 1:**
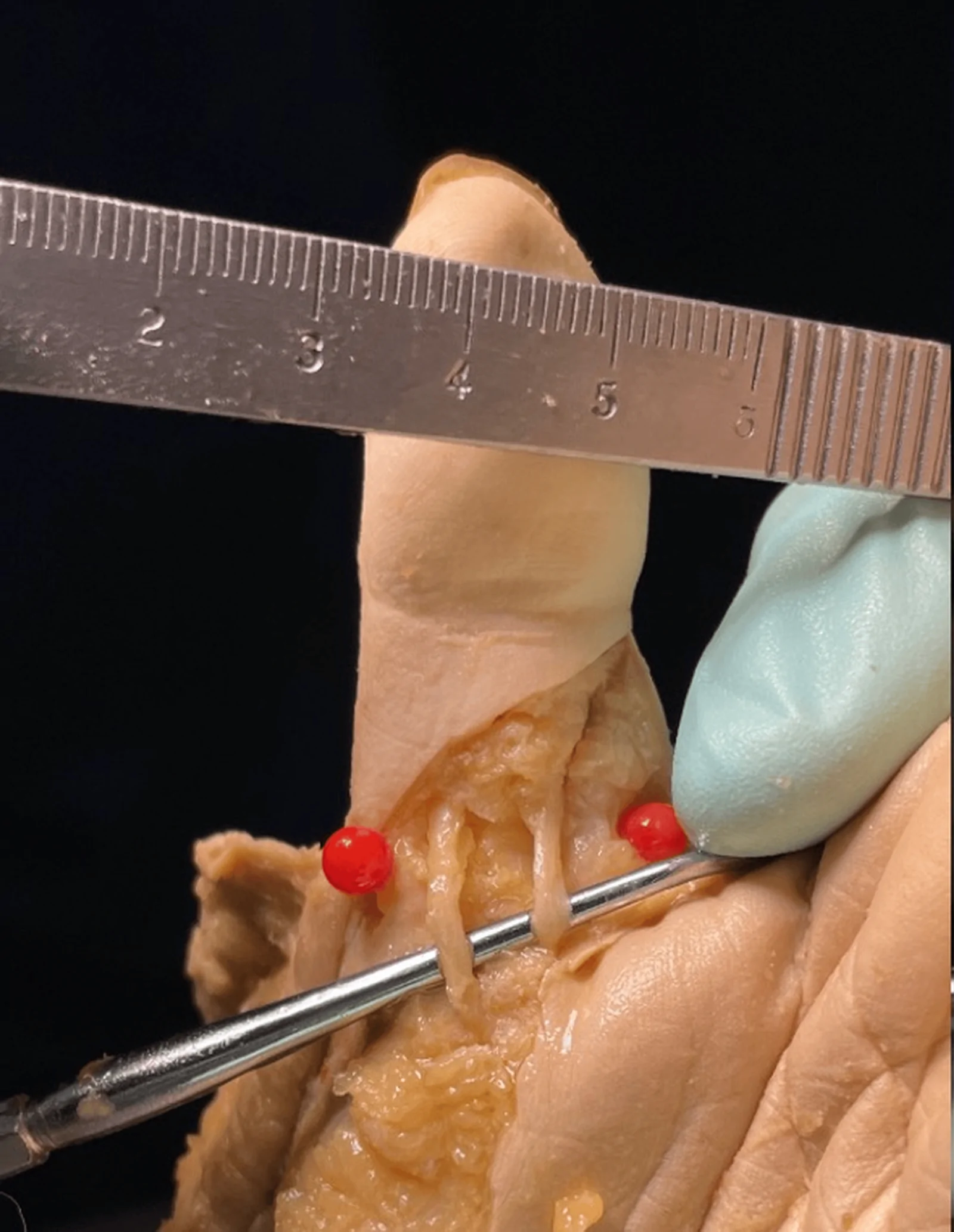
Photograph of atraumatically dissected cadaveric specimen with red pins demonstrating the marked proximal flexion crease and an angled freer isolating both the radial and ulnar digital nerves. Additionally, a scalpel with a standardized engraved ruler was used to assist with accurate measurements.

Each picture included the same scalpel holder, which included a measurement ruler. ImageJ software (NIH, Bethesda, MD) [17] was then utilized to determine the distance from the radial portion of the flexion crease to both the radial and ulnar digital nerves of the thumb. Specifically, the number of pixels from the radial portion of the flexion crease to each digital nerve was obtained. This was then converted to millimeters using the ruler that was uniform and included in each picture. 

Data analysis

Simple statistical analysis was performed using Microsoft Excel (Microsoft® Corp., Redmond, WA) to determine the mean, standard deviation (SD), and range observed between measurements. GraphPad Prism (Version 10.0.0 for Windows, GraphPad Software, Boston, MA, www.graphpad.com) was then used to create figures depicting the number of subjects that had their radial and ulnar digital nerves cross in the radial, middle, or ulnar third portion of the flexion crease.

## Results

This study included 21 unique, soft-embalmed, cadaveric hands. The average length of the hand from the distal wrist flexion crease to the tip of the long finger was 185.21 ± 15.78 mm. The radial digital nerve crossed the crease at 8.38 ± 3.38 mm from the radial aspect of the proximal flexion crease. The ulnar digital nerve crossed the proximal flexion crease of the thumb approximately 16.35 ± 3.80 mm from the radial aspect of the crease. On average, the radial digital nerve crossed at 36.4% ± 9.4% of the distance between the radial and ulnar portions of the proximal flexion crease, while the ulnar digital nerve crossed at approximately 72% ± 8.2%. The radial digital nerve crossed at the radial third of the proximal flexion crease in 43.0% (nine) of patients and in the middle third in 57.0% (12) of patients (Figure 2). The ulnar digital nerve crossed at the ulnar third of the proximal flexion crease in 81.0% (17) of patients and in the middle third in 19.0% (four) of patients (Figure 3).

**Figure 2:**
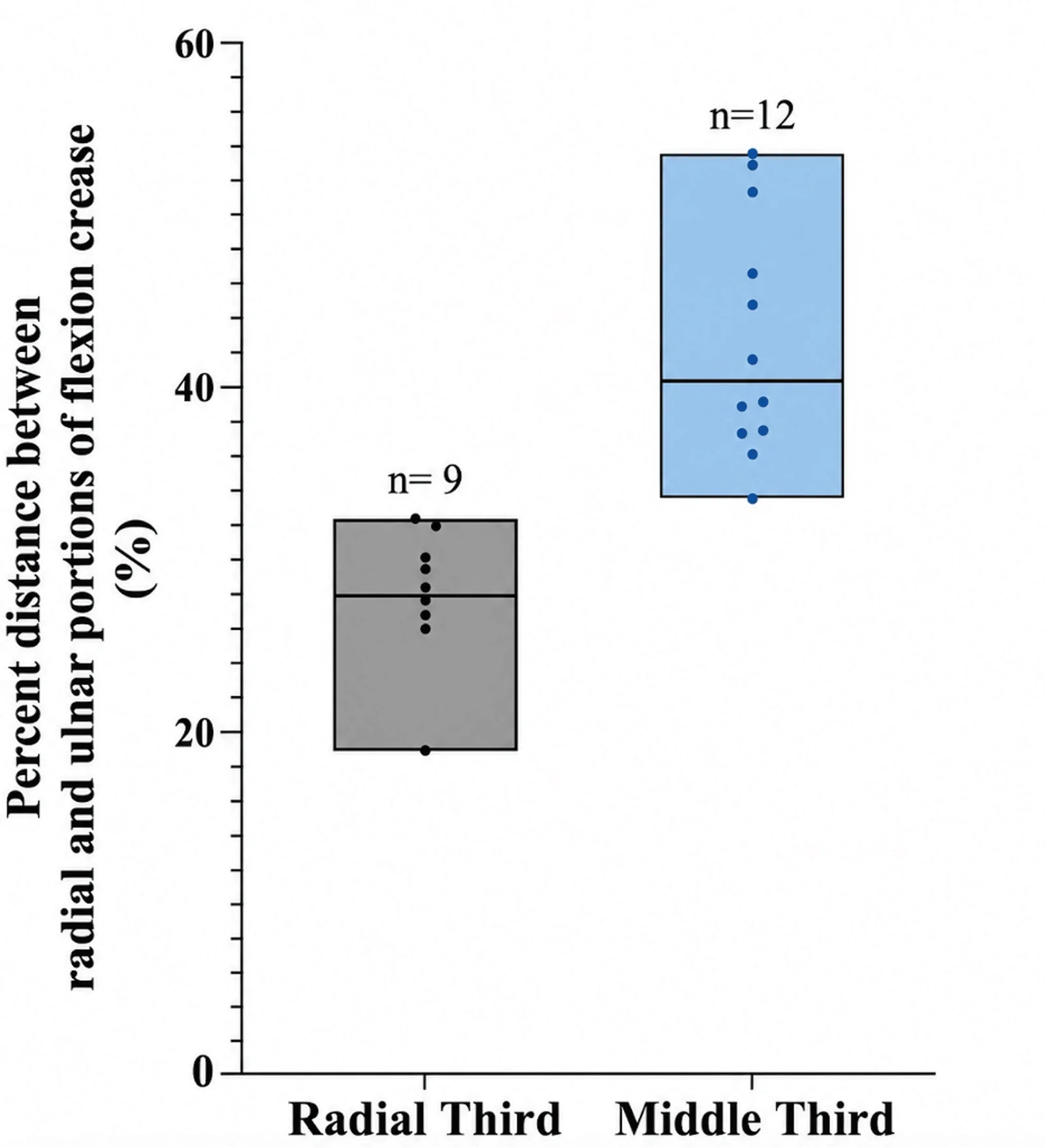
Bar graph demonstrating the percent distance the radial digital nerve crosses between radial and ulnar portions of the flexion crease.

**Figure 3:**
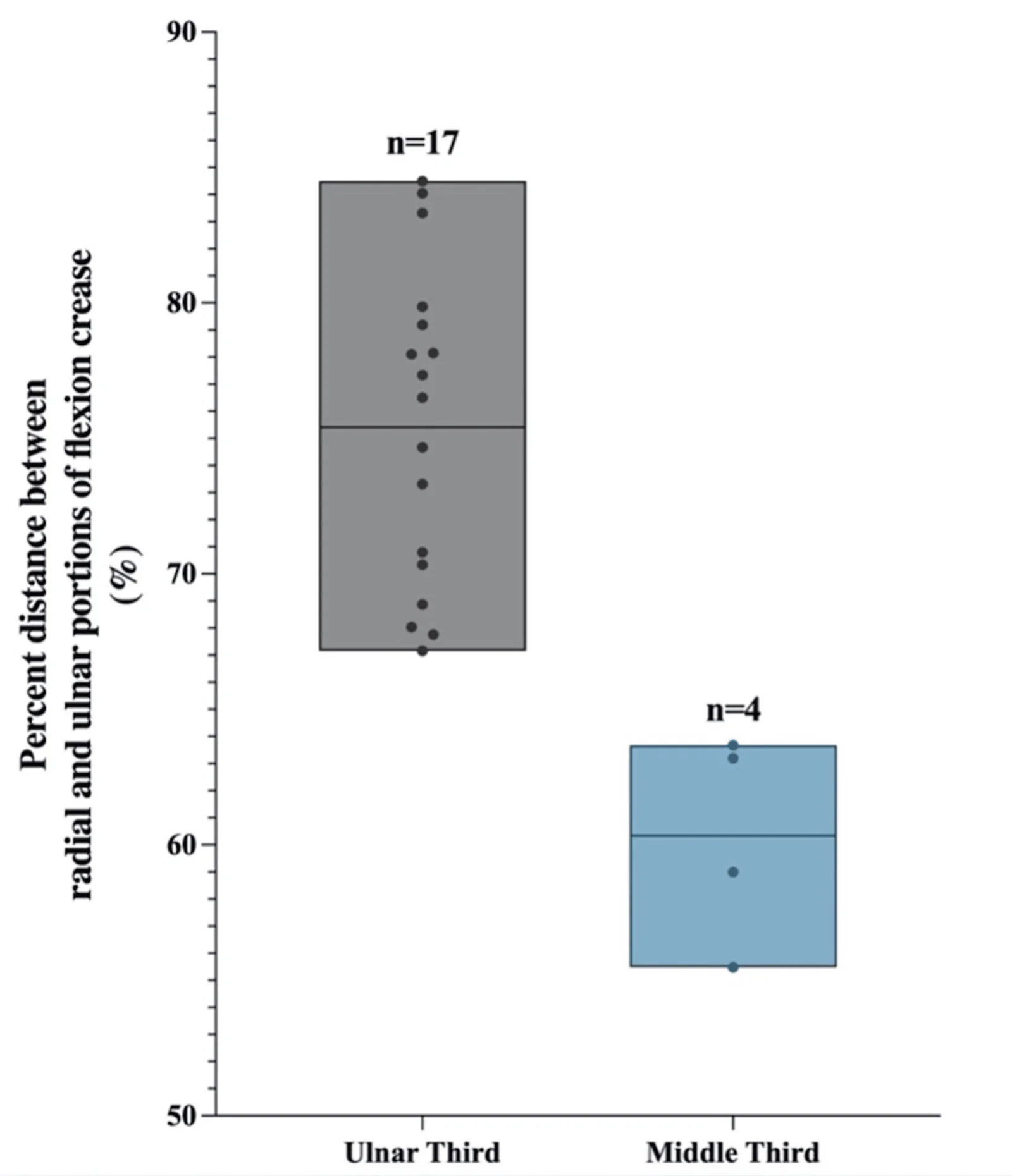
Bar graph demonstrating the percent distance the ulnar digital nerve crosses between radial and ulnar portions of the flexion crease.

## Discussion

The study reveals that the ulnar and radial digital nerves cross at 72.0% and 36.4% of the distance between the radial and ulnar portions of the proximal thumb flexion crease, respectively. In addition to understanding the crossing behavior in relation to the flexion crease, both nerves were found to be traversing distally on the appropriate sides of the flexor pollicis longus tendon. This provides yet another anatomical landmark to reference while performing surgical dissection and retraction.

This information will support surgeons in performing safe procedures near the thumb with minimization of iatrogenic injury to a digital nerve. A recent study by Koopman et al. compares the varying skin incisions used for thumb trigger thumb release and found no statistically significant difference in complications or outcomes based on type of skin incision [18]. Notably, 60% of patients had a “V-shaped” incision, likely due to better visualization to prevent injury. Previous studies have highlighted the association between improper handling of digital nerves to the thumb and postoperative complications, including sensory loss and neuromas [12,19-21]. In the setting of iatrogenic injury to the digital nerves, surgeons should consider multiple factors, the most important of which is the gap size of the nerve defect. In a systematic review and meta-analysis performed by Zhang et al., no nerve repair method was found to have an absolute advantage over the others [20]. The findings contribute to this body of knowledge by providing quantitative data on the distance of both the radial and ulnar digital nerves to the thumb, thereby arming surgeons with valuable information to navigate this anatomically sensitive region more effectively.

While this study provides valuable insights, it is essential to acknowledge its limitations. The sample size may impact the generalizability of our findings. Future research endeavors could explore a larger and more diverse cohort to validate and expand upon our results. Additionally, investigating the functional implications of the observed variations could further enhance our understanding of the digital nerves’ role in thumb innervation.

## Conclusions

This study characterizes the course of the radial and ulnar digital nerves as they cross the proximal thumb flexion crease, providing clinically relevant anatomic landmarks for surgeons. The findings demonstrate that the location of both nerves follows a predictable pattern, with the ulnar and radial digital nerves crossing at 72.0% and 36.4% of the distance between the radial and ulnar portions of the proximal thumb flexion crease, respectively. This can assist in surgical planning and improve intraoperative identification of these structures. By enhancing understanding of thumb digital nerve anatomy, this study offers practical guidance for procedures such as trigger thumb release and other operations in the thenar region, with the goal of reducing iatrogenic nerve injury.

## References

[1] Slutsky DJ (2014). The management of digital nerve injuries. J Hand Surg Am.

[2] Wu K, Aibinder WR, Richards RS, Suh N (2020). A new surface landmark for thumb digital nerve bifurcation: a cadaveric study. J Hand Surg Am.

[3] Tsuchie H, Nishi T, Abe H, Takeshima M, Shimada Y (2013). Trigger finger appearing as gradually increasing digital nerve disorder after surgical treatment. Case Rep Orthop.

[4] Lin GT, Amadio PC, An KN, Cooney WP (1989). Functional anatomy of the human digital flexor pulley system. J Hand Surg Am.

[5] Makkouk AH, Oetgen ME, Swigart CR, Dodds SD (2008). Trigger finger: etiology, evaluation, and treatment. Curr Rev Musculoskelet Med.

[6] Hirasawa Y, Sakakida K, Tokioka T, Ohta Y (1985). An investigation of the digital nerves of the thumb. Clin Orthop Relat Res.

[7] Dębski T, Złotorowicz M, Noszczyk BH (2022). Long-term sensibility outcomes of secondary digital nerve reconstruction with sural nerve autografts: a retrospective study. Eur J Trauma Emerg Surg.

[8] Buldu H, Cepel S, Ki N, Ağritmiş H (2006). References to avoid complications in releases of the trigger thumb: a cadaveric study. Acta Orthop Traumatol Turc.

[9] Aksoy A, Sir E (2019). Complications of percutaneous release of the trigger finger. Cureus.

[10] Sreedharan S, Teoh LC, Chew WY (2011). Neuroma of the radial digital nerve of the middle finger following trigger release. Hand Surg.

[11] Thorpe AP (1988). Results of surgery for trigger finger. J Hand Surg Br.

[12] Carrozzella J, Stern PJ, Von Kuster LC (1989). Transection of radial digital nerve of the thumb during trigger release. J Hand Surg Am.

[13] Guler F, Kose O, Ercan EC, Turan A, Canbora K (2013). Open versus percutaneous release for the treatment of trigger thumb. Orthopedics.

[14] Wallace WA, Coupland RE (1975). Variations in the nerves of the thumb and index finger. J Bone Joint Surg Br.

[15] Bas H, Kleinert JM (1999). Anatomic variations in sensory innervation of the hand and digits. J Hand Surg Am.

[16] Zenn MR, Hoffman L, Latrenta G, Hotchkiss R (1992). Variations in digital nerve anatomy. J Hand Surg Am.

[17] Schneider CA, Rasband WS, Eliceiri KW (2012). NIH Image to ImageJ: 25 years of image analysis. Nat Methods.

[18] Koopman JE, Smit JM, Wouters RM, Selles RW, Michiel Zuidam J, Hundepool CA (2024). Incision techniques for trigger thumb release: a comparison of outcomes of four types of skin incision. J Hand Surg Eur Vol.

[19] Lefebvre R, Russo F, Navo P, Stevanovic M (2020). Characteristics of iatrogenic nerve injury from orthopedic surgery correlate with time to subspecialty presentation. Plast Reconstr Surg Glob Open.

[20] Zhang Y, Hou N, Zhang J (2023). Treatment options for digital nerve injury: a systematic review and meta-analysis. J Orthop Surg Res.

[21] Evertsson L, Björkman A, Turesson C, Arner M, Mellstrand Navarro C (2025). Long-term subjective and objective outcomes after digital nerve repair: a cohort study. J Hand Surg Eur Vol.

